# Protocol to generate a 3D atherogenesis-on-chip model for studying endothelial-macrophage crosstalk in atherogenesis

**DOI:** 10.1016/j.xpro.2024.103559

**Published:** 2025-01-11

**Authors:** Katie M.L. Hanford, Kim E. Dzobo, Miranda Versloot, Jorge Peter, Jeffrey Kroon

**Affiliations:** 1 Department of Experimental Vascular Medicine, Amsterdam UMC, location AMC, Meibergdreef 9, Amsterdam, the Netherlands; 2 Amsterdam Cardiovascular Sciences, Atherosclerosis & Ischemic Syndromes, Amsterdam, the Netherlands; 3 Laboratory of Angiogenesis and Vascular Metabolism, VIB-KU Leuven Center for Cancer Biology, VIB, 3000 Leuven, Belgium; 4 Laboratory of Angiogenesis and Vascular Metabolism, Department of Oncology, KU Leuven and Leuven Cancer Institute (LKI), 3000 Leuven, Belgium

**Keywords:** Cell culture, Flow Cytometry, Immunology, Microscopy, Gene Expression, Antibody

## Abstract

The endothelium is the gatekeeper of vessel health, and its dysfunction is pivotal in driving atherogenesis. Here, we present a protocol to replicate endothelial-macrophage crosstalk during atherogenesis, called the “atherogenesis-on-chip” model, based on the Emulate dual-channel perfusion system. We describe a model for studying endothelial-macrophage interactions during atherogenesis in human aortic endothelial cells and human macrophages using qPCR and secretome analysis, fluorescence microscopy, and flow cytometry. This protocol could be adapted toward more complex plaque microenvironment or other disease settings.

## Highlights

• Steps to develop an organ-on-chip model to study endothelial-macrophage interactions
• Guidance for optimizing immunofluorescence microscopy in organ-on-chip platforms
• Panel design for macrophage phenotyping using flow cytometry
• Strategies for putative receptor-ligand analysis with qPCR and secretome analysis

## Abstract (editor highlights)

Publisher’s note: Undertaking any experimental protocol requires adherence to local institutional guidelines for laboratory safety and ethics.

## Graphical abstract

**Figure:**
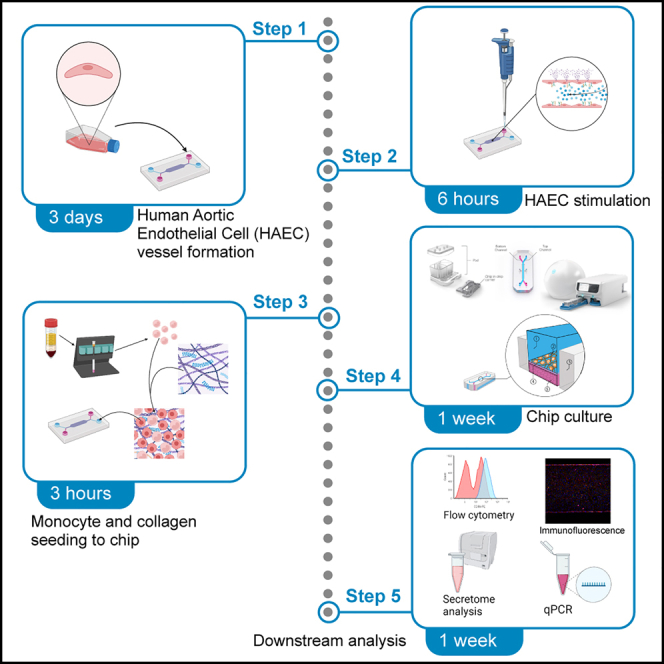

## Before you begin

In this protocol Human Aortic Endothelial Cells (HAECs) are the cell type of choice to mimic the arterial wall. We choose to use freshly isolated CD14^+^ bead isolated monocytes from human peripheral blood mononuclear cells (PBMCs) for their capacity to polarize to macrophages during culture. This method better mirrors the *in vivo* environment compared to cell lines, offering a more physiologically relevant model for our experiments. The protocol could be modified for other endothelial and immune cell types and be expanded by adding other relevant cell types, such as smooth muscle cells (SMCs); however, extensive optimization will be required to tailor the model effectively to these variations. Organ-on-chip culture can be challenging, we therefore urge users to take time to go through the troubleshooting steps of this protocol.

### Background

Cardiovascular diseases (CVDs) are the leading cause of death worldwide, with atherosclerotic ischemic heart disease and stroke being the main cause of CVD morbidity and mortality.^1^ Understanding the complex interplay between the inflamed vasculature and atherosclerotic plaque microenvironment is paramount in the search for new therapeutic targets.

The endothelium is the gatekeeper of vessel health, providing a vital barrier between the arterial lumen and the intima. Endothelial dysfunction has been identified as a common pathway leading to atherogenesis.^2^^,^^3^^,^^4^^,^^5^ During atherogenesis, the endothelium undergoes activation, for example due to oxidized low-density lipoproteins (oxLDL), leading to the secretion of pro-inflammatory cytokines and chemokines, such as IL-6 and MCP-1, and increased expression of the adhesion molecules VCAM-1 and ICAM-1, pivotal for the recruitment of leukocytes to the site of inflammation.^2^ The endothelial barrier also loses its integrity through VE-cadherin internalization, leading to increased permeability and further contributing to the pathogenesis of atherogenesis.^2^ Together, these processes not only facilitate leukocyte recruitment but may also play a crucial role in shifting the subendothelial plaque microenvironment toward a continuous low-grade inflammatory state. Hence, the activated endothelium serves as a key mediator in the progression of atherosclerosis, orchestrating both the initial recruitment of immune cells and maintenance of a pro-inflammatory plaque microenvironment.

Once circulating monocytes have crossed the endothelial barrier into the subendothelial space, they differentiate into macrophages.^6^ Dependent on the signals present in this microenvironment, the macrophages can become polarized into a more pro-inflammatory or more anti-inflammatory state.^6^ Macrophages can also take up oxLDL and become foam cells. The pro-inflammatory factors secreted by pro-inflammatory macrophages and foam cells, as well as activation of the inflammasome in these cells can sustain endothelial activation and its dysfunction.^7^ This, in turn, maintains a low-grade inflammatory state in endothelial cells (ECs), which can further polarize macrophages toward a pro-inflammatory state, thus perpetuating the vicious cycle.

Plaque macrophages can disrupt the extracellular matrix by secreting matrix metalloproteases, leading to increased plaque vulnerability. Given that an activated endothelium and a pro-inflammatory plaque environment are associated with plaque vulnerability and instability,^8^^,^^9^^,^^10^^,^^11^ endothelial-macrophage crosstalk could be a viable target option in atherosclerosis.

In this protocol paper, we demonstrate a working model to study endothelial-macrophage crosstalk in a 3D-vessel-on-chip system, based on the Emulate dual-channel perfusion system. This model can be used to evaluate interventions targeting the endothelium as well as macrophages and assess their effects on macrophage polarization and function. In the future, this model could be expanded to encompass more cell types, such as SMCs or T cells, as well as the addition of circulating and accumulated (oxidized) lipoproteins, such as (ox)LDL, in order to more accurately mimic the complex atherosclerotic plaque environment.

### Institutional permissions

All volunteers who donated blood gave informed consent for the use of their blood in the study, according to the Amsterdam UMC Blood sampling for biomedical experiments (BACON) protocol. The Medical Ethics Committee of the Amsterdam UMC have ruled this study protocol is not subject to the Medical Research Involving Human Subjects Act and no formal approval from the Ethics Committee was required in this instance. However, you will need to acquire informed consent from individuals and comply with your institutional requirements regarding using material from healthy volunteers.

### Media preparation

**Timing: 5 min**

1. Prepare endothelial growth medium (EGM-2) by adding the complete bullet kit to endothelial base medium (EBM-2) (detailed recipe can be found in the materials and equipment section).

### Culturing of human aortic endothelial cells

**Timing: 5–7 days**

***Note:*** HAECs cells can be cultured until approximately passage 7 (p7) before they lose their capacity to proliferate.

**CRITICAL:** While in culture, HAECs should always maintain confluence between 25% (seeding density) and 80% (passage density). If seeded too sparsely HAECs, will not proliferate as they require signals from neighboring cells. HAECs are only allowed to reach 100% confluence once they are seeded in the chip, as they will no longer proliferate once they become contact-inhibited.

2. Thaw a HAEC ampoule, containing approximately 1 × 10^6^ cells.
  a. Coat a T75 flask with 3 mL 3.125 μg/mL fibronectin (FN) and incubate for at least 60 min at 37°C, 5% CO2, then remove the FN from the T75 flask.
    ***Note:*** The flask does not have to be washed before adding HAECs.
  b. Partially thaw HAEC in a 37°C water bath.
  c. Quickly add cell suspension to 8 mL of cold EGM-2 and seed cells in the FN coated T75 flask.
  d. Wash the vial of HAECs one time with 2 mL regular EGM-2 to ensure removal of all cells from the vial, add this to the T75.
  e. Evenly distribute the cells by gently tipping the T75 flask crosswise. Incubate at 37°C, 5% CO2.
  f. Once cells have attached, which occurs after 6–8 h, refresh with 10 mL EGM-2 medium at 37°C (from here on warm EGM-2) to completely remove the DMSO.
3. When HAEC reach 90% confluence they can be passaged.
  a. Coat 3 T75 flasks (3 mL of FN per flask) and incubate for at least 60 min at 37°C, 5% CO2.
  b. Remove medium from the T75 flask that contains your 90% confluent HAECs.
  c. Wash the HAECs 2x with 5 mL PBS. Swirl gently.
  d. Add 2 mL of room temperature (18°C–21°C) accutase to the T75 that contains the HAECs to detach the cells. Incubate for approximately 2 min at 37°C. Confirm that the cells are detached using a microscope with 10x objective to get a full overview of the flask.
  e. Once all HAECs are detached, add 7 mL warm EGM-2 to create a cell suspension of 9 mL.
  f. Spin cells at 500 g, 5 min at 21°C.
  g. Meanwhile, remove FN from the previously coated T75 flasks (step 3a) and replace with 7 mL of warm EGM-2.
  h. Aspirate the supernatant and resuspend the cell pellet in 9 mL warm EGM-2.
  i. Split the 9 mL of cell suspension equally over the three T75 flasks. The end volume should be 10 mL, for culturing over the weekend add an extra 3 mL of warm EGM-2.

### Preparation of chips—Activation and coating

**Timing: 1 h**

This section is an abbreviated version of the Chip Preparation found in the Chip-S1 Basic Research Kit Protocol explaining how to prepare the chips prior to chip culture. The full protocol can be found on the Emulate website.

**CRITICAL:** Throughout chip preparation avoid bubbles within chip channels as this is critical for success of the activation and coating of the chips.

***Note:*** The activation solution must be used immediately after preparation. It is therefore recommended to activate as many chips as possible and then store activated chips with sterile PBS at 4°C.

4. In the biosafety cabinet (BSC), align all chips facing the same direction in a 150 mm dish. Ensure that each chip carrier is clearly labeled with permanent marker.
5. Turn off the light in the BSC, as the activation solution is light sensitive.
6. Make the activation solution by mixing 1 vial of ER-1 powder with 10 mL of ER-2 solution for a working concentration of 0.5 mg/mL.
7. Activate chips by pipetting activation solution into both chip inlets. Aspirate spill-over from the outlets using a tissue. 200 μL will fill roughly 3 chips.
8. Activate the chips by placing in a UV light box for 10 min.
9. Wash chips twice with ER2 solution and repeat the activation steps 7 and 8.
10. After the second UV activation, wash each channel of the chips twice with ER2 solution and then once with cold PBS.
11. The light in the BSC may be on from this point forward, as following UV activation there is no light sensitive material.

**Pause point:** Activated chips can be stored at 4°C with sterile PBS in both channels and PBS filled filter tips inserted into all inlet and outlet ports. Empirical experiments have shown successful storage for 3 months.

12. Prepare FN at a concentration of 30 μg/mL, this will be used to coat the bottom vascular channel.
13. Fully aspirate the cold PBS from the bottom channel, while leaving PBS in the top channel, this channel will be used for the macrophage collagen gel mixture later.
14. Carefully introduce 50 μL FN solution through the bottom channel inlet until a small FN droplet forms on top of the outlet port.
15. Add a droplet of FN above the inlet and outlet port of the bottom channel and a droplet of PBS on the inlet and outlet of the top channel.
16. Inspect channels under the microscope to ensure that no bubbles are present within the channels.

***Note:*** If bubbles are present, dislodge by washing the channel with FN solution until all bubbles have been removed. If bubbles persist refer to the troubleshooting steps provided by Emulate in the Chip-S1 Basic Research Kit Protocol these troubleshooting steps are also detailed at the end of this protocol under the troubleshooting heading.

17. Add 1.5 mL of PBS to the cap of a 15 mL conical tube. Place the PBS cap in the 150 mm culture dish with the chips to provide extra humidity and place the lid on the dish.

**Pause point:** Coated chips can be stored at 4°C for up to 1 week.

18. Place FN-coated chips in an incubator (37°C, 5% CO_2_) 1 h prior to cell seeding.

## Key resources table

**Table:** 

REAGENT or RESOURCE	SOURCE	IDENTIFIER
**Antibodies**		

Mouse monoclonal IgG_1_ anti-VE-cadherin (F-8) 1:100	Santa Cruz	SC-9989 RRID:AB_2077957
CD64 recombinant rabbit monoclonal antibody (27) 1:100	Invitrogen, Thermo Fisher Scientific	MA5-29706 RRID:AB_2785530
CD68 antibody [KP1] 1:100	Abcam	Ab955 RRID:AB_307338
CD68 monoclonal antibody (514H12)	Invitrogen, Thermo Fisher Scientific	MA1-80133 RRID:AB_929283
Goat anti-mouse IgG1 Alexa Fluor 647 1:200	Invitrogen, Thermo Fisher Scientific	A21240 RRID:AB_2535809
Goat anti-rabbit IgG (H + L) Alexa Fluor 488 1:200	Thermo Fisher Scientific	A-11034 RRID:AB_2576217
Goat anti-mouse IgG2a cross-adsorbed secondary antibody, Alexa Fluor 568	Thermo Fisher Scientific	A-21134 RRID:AB_2535773
APC-H7 mouse anti-human CD14 1:100	BD Biosciences	641394 RRID:AB_1645725
PerCP-Cy5.5 mouse anti-human HLA-DR 1:200	BD Biosciences	560652 RRID:AB_1727529
PE-Cy7 mouse anti-human CD11b 1:100	BD Biosciences	561685 RRID:AB_10893799
PE mouse anti-human CD80 1:100	BD Biosciences	557227 RRID:AB_396606
FITC mouse anti-human CD86 1:100	BD Biosciences	555657 RRID:AB_396012
APC anti-human CD64 antibody 1:100	BioLegend	305013 RRID:AB_1595539
IL-1R1 antibody 1:1,000	Santa Cruz	SC-393998 RRID:AB_2737063
VCAM-1 (E1E8X) antibody 1:1,000	Cell Signaling Technology	13662S
Beta-actin (GT5512) 1:4,000	GeneTex	GTX629630 RRID:AB_2728646
Peroxidase-conjugated goat anti-mouse immunoglobulins 1:5,000	Dako	P0447
Peroxidase-conjugated goat anti-rabbit immunoglobulins 1:2,000	Dako	p0448

**Biological samples**		

Human whole blood	Amsterdam UMC, location AMC	N/A

**Chemicals, peptides, and recombinant proteins**		

EBM-2 basal medium	Lonza	CC-3156
EGM-2 endothelial cell growth medium-2 BulletKit	Lonza	CC-3162
Fibronectin bovine plasma	Sigma-Aldrich	F1141-1MG
Collagen type I, rat tail	ibidi	50201
Collagenase type I	Gibco	17100017
CD14 MicroBeads	Miltenyi Biotec	130-050-201
Lymphoprep	STEMCELL Technologies	07851
Lipofectamine RNAiMAX transfection reagent	Invitrogen	13778150
Opti-MEM	Gibco	11058021
IL-1β	Sigma-Aldrich	SRP6169
Hoechst 1:1,000	Thermo Fisher Scientific	62249
DAPI 1:5,000	BioLegend	422801
RPMI 1640 + GlutaMAX	Gibco	61870036
Accutase	Merck	A6964
PBS	Gibco	10010–015
Formaldehyde solution	Sigma-Aldrich	F8775
Bovine serum albumin	Sigma-Aldrich	A3912
Triton X-100	Sigma-Aldrich	T8787
Trizol	Invitrogen	15596018
SensiFAST SYBR No-ROX Kit	GC Biotech	Bio-98020
UltraPure 0.5 M EDTA	Invitrogen	15575020
30% Bovine serum albumin solution	Sigma-Aldrich	A9576
DMEM	Gibco	41966–029
Fetal bovine serum	Capricon Scientific	12A
Collagenase type I	Gibco	17100017
HEPES (1 M)	Gibco	15630–056

**Critical commercial assays**		

ProteomeProfiler Human XL Cytokine Array Kit	R&D Systems	ARY022B
Chip-S1 Basic Research Kit (including ER-1, ER-2, S1 chips and Pods)	Emulate	BRK-S1-WER-12

**Experimental models: Cell lines**		

Human aortic endothelial cells	Lonza	CC-2535

**Oligonucleotides**		

siRNA silencer: IL1R	Thermo Fisher Scientific	106303
***IL1R*** : forward: TGGAAGTGGAATGGGTCAGT, reverse: TTGCAGGATTTTCCACACTGTAAT	Designed in-house	N/A
***IL6*** : forward: CTG CAG AAA AAG GCA AAG AAT CTA, reverse: GTT GTC ATG TCC TGC AGC C	Designed in-house	N/A
***VCAM1***: forward: TGT CAA TGT TGC CCC CAG A TGC TCC, reverse: ACA GGA TTT TCG GA	Designed in-house	N/A
All other primers see Tables 4 and 5	Designed in-house	N/A

**Software and algorithms**		

GraphPad Prism v.10	GraphPad	https://www.graphpad.com/features
Las-X	Leica	https://www.leica-microsystems.com/products/microscope-software/p/leica-las-x-ls/
FlowJo v. 10	BD Biosciences	https://www.flowjo.com/
BD FACSDiva	BD Biosciences	https://www.bdbiosciences.com/en-nl/products/software/instrument-software/bd-facsdiva-software
CFX Maestro	Bio-Rad	https://www.bio-rad.com/en-nl/product/cfx-maestro-software-for-cfx-real-time-pcr-instruments?ID=OKZP7E15
Fiji	ImageJ	https://imagej.net/software/fiji/
Imaris v.10.1.1	Oxford Instruments	https://imaris.oxinst.com/
ImageLab	Bio-Rad	https://www.bio-rad.com/en-nl/product/image-lab-software?ID=KRE6P5E8Z
Biorender	BioRender	https://www.biorender.com/
R4.4.1	R-project	https://www.r-project.org/
RStudio 2024.04.02	R 4.4.1	https://posit.co/download/rstudio-desktop/
ggplot2 3.5.1	CRAN	https://cran.r-project.org/
pheatmap 1.0.12	CRAN	https://cran.r-project.org/

**Other**		

Zoë-CM1 Culture Module	Emulate	N/A
Orb-HM1 Hub Module	Emulate	N/A
Steriflip- HV filters	EMD Millipore	SE1M003M00
UV light Box	Emulate	N/A
Chip cradle	Emulate	N/A
4 mm diameter discs of 50 μM thick PET	Emulate	N/A
Revolve microscope	Echo	N/A
Thunder microscope	Leica	N/A
OctoMACS separator	Miltenyi Biotec	130-042-108
MICA microscope	Leica	N/A
MS column	Miltenyi Biotec	130-042-201
Leucocep tubes	Greiner Bio-one	227290
BD FacsCantoII	BD Biosciences	N/A
pH strips 2.0–9.0	Merck	1.09584.0001
10 mL EDTA Vacutainer blood collection tubes	BD	367525
Vacuum pump that can reach −70 kPa	N/A	N/A

## Materials and equipment

**Table:** EGM-2 media

Reagent	Final concentration	Amount
EBM-2 Endothelial Cell Growth Basal Medium-2	N/A	500 mL
Fetal Bovine serum	2%	10 mL
Hydrocortisone	N/A	0.2 mL
hFGF-B	N/A	2 mL
VEGF	N/A	0.5 mL
R3-IGF-1	N/A	0.5 mL
Ascorbic Acid	N/A	0.5 mL
hEGF	N/A	0.5 mL
GA-1000	N/A	0.5 mL
Heparin	N/A	0.5 mL
**Total**	**N/A**	**515.2 mL**

Complete EGM-2 can be stored for up to 3 months at 4°C.

**Table:** Digestion solution

Reagent	Final concentration	Amount
DMEM	N/A	14.46 mL
Fetal Bovine serum	10%	1.5 mL
Collagenase type I	1 mg/mL	1.5 mg
HEPES (1 M)	25 mM	40 μL
**Total**	**N/A**	**15 mL**

Digestion solution can be stored for 1 month at 4°C.

• PBS-EDTA: add 2 mL sterile EDTA (0.5 M) to 500 mL sterile PBS for 2 mM PBS-EDTA.

 PBS-EDTA can be stored for 3 months at 4°C.

• MACS buffer: add 0.5 mL 30% BSA to 29.5 mL PBS-EDTA.

 MACS buffer can be stored for 2 weeks at 4°C.

• FACS buffer: add 0.5 g BSA to 50 mL PBS-EDTA.

 FACS buffer can be stored for 2 weeks at 4°C.

• 0.1% Triton X-100: diluted 100 μL Triton X-100 in 100 mL PBS.
• 5% BSA: add 2.5 g BSA to 50 mL PBS.

 5% BSA can be stored for 2 weeks at 4°C.

• 1% BSA: dilute 5% BSA 5x in PBS.

 1% BSA can be stored for 2 weeks at 4°C.

## Step-by-step method details

### Endothelial cell seeding to chip

**Timing: 5 h total. Chip seeding: 1 h. Cell attachment: 4 h.**

This step describes the seeding of the endothelium to the chip to create the ‘blood vessel’ compartment of the atherogenesis-on-chip model. The cell seeding technique is largely the same as described by Emulate in the Chip-S1 Basic Research Kit Protocol (https://emulatebio.com/). An abbreviated protocol is provided below.

**CRITICAL:** To ensure uniform chip seeding it is vital to pipette in one smooth motion. Additionally, bubbles should be avoided. If bubbles occur, cells need to be reseeded.

1. HAECs that have been expanded in culture must be harvested and counted for channel seeding.

***Note:*** For details on how to detach HAECs see the previous section culturing of human aortic endothelial cells.

2. Once endothelial cells have been harvested spin them down at 500g, 5 min at 21°C.
3. Meanwhile, remove FN and PBS from the chip channels and wash both channels once with warm EGM-2. Leave EGM-2 in the channels.
4. Carefully aspirate the supernatant, leaving approximately 100 μL of medium above the cell pellet.
5. Loosen the cell pellet by gently resuspending with a p200.
6. Using a P200 pipette, gently re-suspend the cells by adding 200 μL of EGM-2.
7. Count the cells using your preferred cell counting method, for example with a hemocytometer or automated cell counter.
8. Dilute the endothelial cells to the desired seeding density in EGM-2. In this case 10 × 10^6^/mL when using HAECs at p5-p6.

**CRITICAL:** Gently agitate cell suspension using a p200 before seeding each chip to ensure a homogeneous cell suspension.

9. Seed 20 μL of the endothelial cell suspension (200,000 cells) into the bottom channel of one chip first, while aspirating the outflow.

**CRITICAL:** To ensure uniform chip seeding it is vital to pipette in one smooth motion.

**CRITICAL:** While aspirating the outflow, the aspirator should be kept at a distance from the outlet port. If the aspirator is held too close to the outlet port the HAECs will be sucked out of the channel.

10. Cover the dish and transfer to the microscope to check the seeding density within the chip.
11. After confirming the correct cell density, seed cells in the remaining chips.
12. Invert each chip and rest the edge of the chip carrier on the chip cradle.
13. Place the chips, still in the dish, in the incubator (37°C with 5% CO_2_) for approximately 2–4 h, or until cells in the bottom channel have started to attached, this can be confirmed by microscope.
14. Once endothelial cells have started to attached flip the chips back to an upright position in the dish.

***Note:*** If desired, when flipping back to the upright position a fresh 20 μL of HAEC suspension (200,000 cells) can be seeded into the bottom channel and left to attach. This will reduce the number of days needed for the bottom channel to form a confluent vessel.

15. Perform a gravity wash on the chips by gently dropping 200 μL on top of both inlet ports of top and bottom channels. This should cause medium to gently flow through the channel, spilling out of the outlets.

***Note:*** Although there are no cells in the top channel, the gravity wash should also be performed for this channel in order to provide extra nutrients to the cells in the bottom channel.

***Note:*** If the media does not flow through the channel, very gently pipette a small amount of medium into the inlets, until a small droplet appears on the outlet, or until a bubble is ejected through the outlet.

**CRITICAL:** Make sure there are no bubbles in either channel during chip culture as these bubbles will affect nutrient availability and endothelial cell viability.

**Figure 1:**
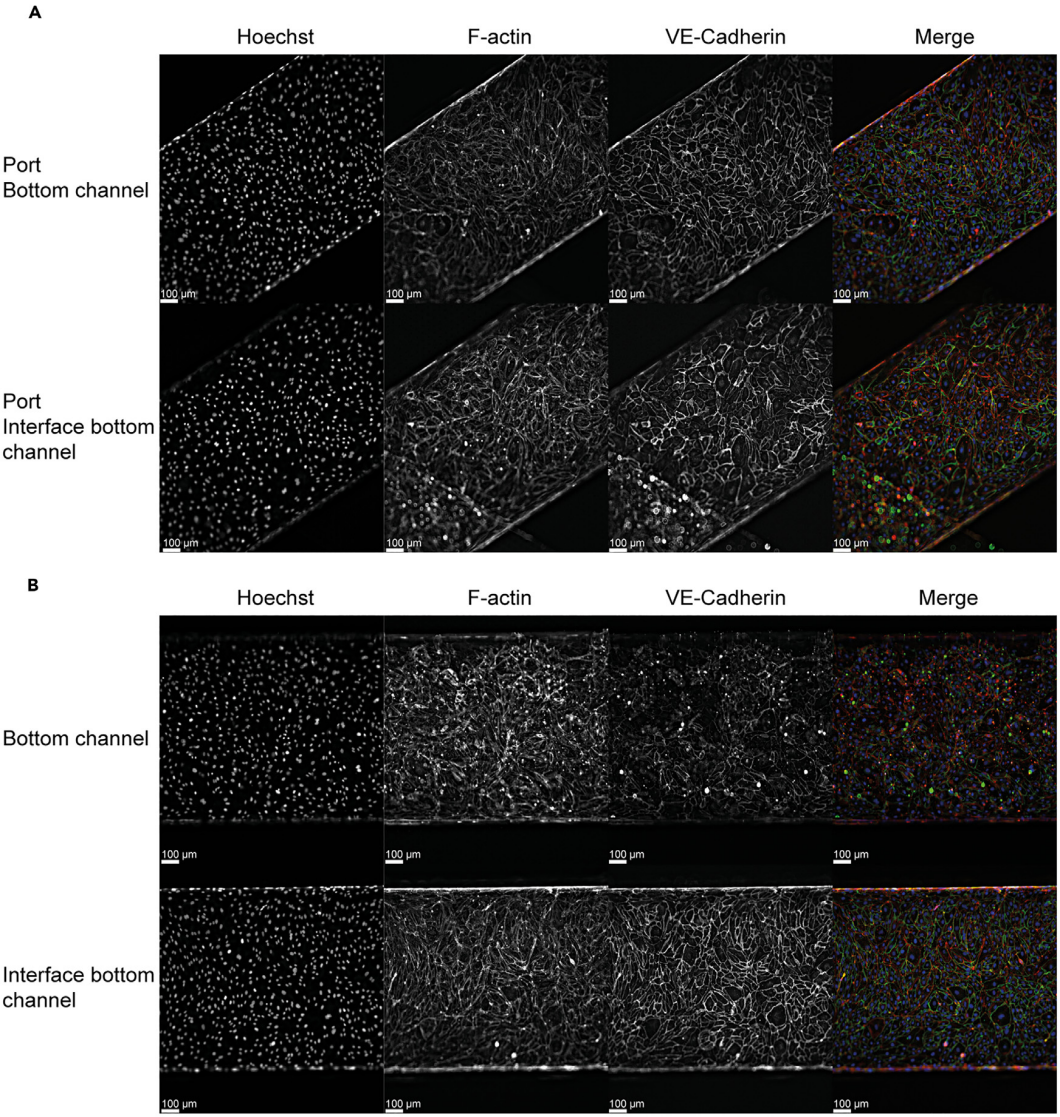
At day 3 a confluent blood vessel channel is formed Representative immunofluorescence images of chip channels. (A) Single channel and overlay image of the bottom channel inlet stained for Hoechst (blue), F-actin (red) and VE-Cadherin (green). (B) Single channel and overlay image of the bottom channel stained for Hoechst (blue), F-actin (red) and VE-Cadherin (green). Scale bars 100 μm. *N* = 4.

16. Place additional droplets of media to fully cover all inlet and outlet ports to prevent evaporation from the ports.
17. Incubate chips in the incubator (37°C, 5% CO_2_).
18. Repeat the gravity wash daily to refresh the medium.
19. 3 days post seeding the vessel channel should be fully confluent (Figure 1).

### Endothelial cell stimulation

**Timing: 6 h**

Once the bottom vessel channel is fully confluent the endothelial cell stimulation can be performed.

20. Wash chips with warm EGM-2, while aspirating the outflow.
21. Plug outlet ports of both channels with filtered 200 μL tips.

**CRITICAL:** Make sure there are no air bubbles between the tip and the ports, as this prevents medium flow/nutrient exchange in the port.

22. Add 200 μL warm EGM-2 to the top channel through the inlet, leaving the filtered 200 μL tip in the inlet port.

***Note:*** The tips are left in the ports to ensure there is no evaporation of medium during the stimulation.

23. Add 200 μL EGM-2 ± IL-1b (1 ng/ml) to the bottom endothelial channel, leaving the 200 μL tip in the inlet port.
24. Place in the incubator (37°C, 5% CO_2_) for 6 h.
25. After 6 h remove all tips and wash the chips twice by pipetting 200 μL warm EGM-2 through the inlets of both channels while aspirating the outflow or by performing a gravity wash.
26. Leave warm EGM-2 in the chip channels and add media droplets on the inlet and outlet ports and place chips in the incubator (37°C, 5% CO_2_) until ready for monocyte/gel seeding.

### Monocyte isolation

**Timing: 3 h**

This section describes the isolation of monocytes from whole blood. Alternatively, your own standard monocyte isolation protocol can be used. Perform this part of the protocol while the chips are being stimulated for 6 h with IL-1β.

27. Collect 2x 10 mL EDTA blood from a donor who has given informed consent.
28. Add 15 mL Lymphoprep to 2x 50 mL Leucosep tubes.
29. Spin down at 1000g for 30 s at 21°C with maximum brake.
30. Distribute the blood over 2x 50 mL falcon tubes.
31. Rinse empty blood tubes 2x with PBS-EDTA (±4 mL) and add the residual blood to the falcon tubes.
32. Dilute the blood with PBS-EDTA to an end volume of 35 mL.
33. Add 35 mL diluted blood into 50 mL Leucosep tube (containing lymphoprep).
34. Place tubes in centrifuge buckets in balance. Centrifuge at 1000g for 15 min without brake (acceleration 9 – deceleration 1) at 21°C.
35. Remove ∼15 mL of the plasma layer carefully using a 10 mL pipette.
36. Pour off the PBMCs into 50 mL falcon tubes (PBMCs from one tube are placed in 1 new tube).
37. Adjust the volume to 50 mL with cold PBS-EDTA.

***Note:*** From now onwards work on ice.

38. Spin 300g for 10 min at 4°C.
39. Remove the supernatant using a vacuum system or decanting.
40. Resuspend the cell pellets and pool in one 50 mL falcon tube.
41. Wash with cold PBS-EDTA (add PBS-EDTA up to 50 mL).
42. Count cells using your preferred counting method.
43. Spin 300g for 10 min at 4°C.
44. Remove the supernatant by decantation, removing as much PBS-EDTA as possible with a P1000.
45. Resuspend in 40 μL MACS buffer per 10 × 10^6^ cells.
46. Add 10 μL of CD14 beads per 10 × 10^6^ cells.
47. Mix well, incubate for 15 min in refrigerator (2°C–8°C) or on ice.
48. Wash cells by adding 1 mL MACS buffer per 10 × 10^6^ cells.
49. Spin 300 g for 10 min at 4°C.
50. Aspirate supernatant completely.
51. Resuspend up to 100 × 10^6^ cells in 500 μL MACS buffer.
52. Place MS column in magnetic separator (keep plunger sterile).
53. Rinse the column with 500 μL MACS buffer.
54. Apply cell suspension in column reservoir (500 μL).
55. Wash column with 500 μL MACS buffer. Only add buffer once column reservoir is empty.
56. Repeat wash twice more for a total of 3 washes.
57. Remove column from separator, and place on suitable collection tube (15 mL tube).
58. Pipette 1 mL MACS buffer onto column reservoir, and flush out the magnetically labeled cells by firmly pushing the plunger in the column.
59. Spin 300 g for 10 min at 4°C, remove MACS buffer (with P1000), take up cells in 1–3 mL RPMI with glutaMAX.
60. Count cell using your preferred counting method.
61. Resuspend monocytes to a concentration of 10 × 10^6^/mL in in RPMI with glutaMAX.

### Collagen gel preparation

**Timing: 15 min**

This section describes the preparation of the collagen monocyte mixture. This gel mixture simplistically mimics the ‘plaque’ compartment in the top channel of the chip.

**CRITICAL:** Collagen polymerizes quickly above 10°C. Therefore, it is essential to perform all steps on ice and with frozen tips, racks, Eppendorf tubes and any other equipment you may use.

62. Mix collagen and RPMI to a concentration of 3 mg/mL.
63. Neutralize collagen by adding 4 μL 1N NaOH, adjusting to around pH 8. Add an additional 1 μL at a time to reach pH 8 if required.
64. Make gel/cell mixture on ice by adding equal volumes of neutralized collagen and monocyte suspension.

### Plaque seeding to top channel

**Timing: 1.5 h total**

**Timing: 30 min for steps 65–72**

This section describes the seeding of the collagen gel and monocyte mixture to the top channel.

**CRITICAL:** Work with one chip at a time, always keeping the collagen gel-cell mixture on ice at all times.

65. Aspirate all liquid from both chip channels. Channels should be dry.
66. Loosely plug both top and bottom chip outlets with filtered pipet tips, ensuring that the tips are not pressed too deep into the ports to allow for liquid flow.
67. Pipet 70 μL of the gel-cell mixture (350,000 monocytes) into the top channel inlet, until liquid just starts to rise into the tip in the outlet. Leave the tip in the inlet port.
68. Inspect the chip, by eye, for bubbles trapped within the gel.
69. If there are visible bubbles within the gel, quickly aspirate the gel-cell mix and fill again.
70. In the same manner as the gel channel, fill the bottom channel with 70 μL of EGM-2, leaving the tip in the inlet port.
71. The media and gel levels in all tips should be approximately equal.
72. Repeat step 65–71 for all chips.
73. Place the chips in the incubator (37°C, 5% CO_2_) for 1 h to allow the gel to polymerize.

### Preparation of chips for organ-chip culture in Zoë module

**Timing: 5 h 45 min. Gas equilibration of media: 1 h 10 min. Prime Pods: 5 min. Wash Chips and PET disc placement: 10 min. Chip connection to Pods: 5 min. Running the Regulate cycle: 4 h 15 min.**

This section provides a brief overview of the steps needed to prepare the chips for culturing in the Zoë module. The full details, including diagrams and explanations of the Pod components can be found in the Chip-S1 Basic Research Kit protocol on the Emulate website (https://emulatebio.com/).

**CRITICAL:** Gas equilibration of the media is critical for the success of organ-chip culture as it reduces the chance of bubble formation in the chip and/or Pod. Minimize the time the medium is outside of a warmed environment as gas equilibrium can become compromised when the medium is allowed to cool.

74. Gas equilibration of media.
  a. Warm EGM-2 in a 50 mL conical tube at 37°C in a water or bead bath for at least 1 h. At least 4.5 mL of medium is needed per chip.
  b. Connect the 50 mL tube with warmed medium to a Steriflip unit, apply vacuum to remove bubbles.
  c. Leave the filtered medium under vacuum, then place the conical tube containing media in the incubator (37°C, 5% CO_2_) with the cap loose.
75. Prime pods.
  a. Working in the BSC place the Pods into the Zoë trays.
  b. Add 1 mL of media to the Pod outlet reservoir corresponding to the top gel channel. This will ensure that the gel remains hydrated.
  c. Pipette 3 mL of pre-equilibrated, warm media to the inlet reservoir corresponding to bottom blood vessel channel.
  d. Also pipette 500 μL of pre-equilibrated, warm media to the outlet reservoir of the bottom blood vessel channel, directly over the outlet Via.
  e. Place the trays with Pods into the Zoë and run the Prime cycle twice.
  f. Verify that the Pods were successfully primed by inspecting the underside of the Pod. There should be small droplets of media at the ports where media was added. If any Pod does not show droplets, re-run the “Prime” cycle on those Pods.

***Note:*** As the gel-filled channel will not have any flow-through a PET disc is placed on the inlet of this channel.

76. Wash chips and place PET discs.
  a. In the BSC, remove all the pipette tips from the chips and perform a gravity wash on the bottom channel only.
    ***Note:*** If any bubbles are observed in the bottom channel, clear them by flushing the channel with equilibrated media. If small bubbles are observed in the gel channel ports or in the gel itself, do not attempt to remove them; these will be removed during the Regulate cycle.
  b. Then add droplets to all four ports.
  c. Just before attaching the chip to the Pod, place a 4 mm diameter disc of 50 μm thick PET onto the gel-filled channel inlet with a pair of tweezers, taking care to center the disc over the inlet port.
  d. Depress the PET disc firmly onto the chip and remove the excess media from the droplet by carefully aspirating excess media next to the inlet port.
  e. Do not remove droplets from the outlet ports of the chip.
77. Connect chips to pods
  a. Slide the chip carrier into the tracks on the underside of the Pod until the chip carrier has seated fully.
  b. Gently, but firmly, depress the chip tab in and up to engage the tab of the chip carrier with the Pod.
  c. Aspirate any excess medium on the chip surface from the Pod window and ensure the PET disk is still in position.
  d. Place the Pod with connected chip onto the tray, confirm that there is sufficient media in the appropriate Pod inlet and outlet reservoirs and that the Pod lids are flat and secure.
  e. Place trays that are holding Pods and chips immediately into Zoë to prevent media from cooling and losing its gas equilibration.
78. Program the appropriate Organ-Chip culture conditions on Zoë.
  ***Note:*** These conditions will start as soon as the Regulate cycle is complete.
  a. The flow rate for the gel-filled channel must be set a 0 μL/h.
  b. The flow rate for the perfusion channel can be set at 10 μL/h. No stretch.
79. Run the Regulate cycle. The Regulate cycle lasts 2 h.
80. When the Regulate cycle is complete, flush the pod vias (where medium flows from the pod to the chip) of the bottom channel.
  a. Using media within the pod reservoir, pipette 200 μL of media directly over the top of the via to dislodge any bubbles that may be present.
81. Run a second Regulate cycle.

**Pause point:** The Via flush and second Regulate cycle can also be performed the next day.

### Sampling, media replenishment, and addition of stimuli

**Timing: 3 h 10 min**

This step is an abbreviated version of that described by Emulate in the Chip-S1 Basic Research Kit Protocol (https://emulatebio.com/) on how to collect media for use in downstream readouts such as ELISA and how to replenish media/stimulate the cells.

In this protocol sampling and addition of stimuli are performed 3 days post chip connection.

82. Pause the Zoë, remove the trays and place in the BSC.
83. Inspect each chip for bubbles by eye and assess cell viability using a microscope. If desired capture representative images at 10X or 20X magnification.
84. Collect effluent medium from Pod outlet reservoirs for analysis, discard excess effluent medium.

**CRITICAL:** Ensure that a thin liquid film still covers the reservoir vias so that no air is introduced into the vias.

85. Refresh the media reservoirs with fresh, warm, equilibrated medium. At this point add warm equilibrated EGM-2 ± 1 ng/mL IL-1β to the inlet reservoir.
86. Perform a via wash, then return the trays of Pods to the Zoë and resume chip culture with the same culture conditions.
87. Stimulate the chips for 3 h.
88. Pause the Zoë, remove the trays and place in the BSC.
89. Collect effluent medium from Pod outlet reservoirs for analysis, discard excess effluent medium.

**CRITICAL:** Ensure that a thin liquid film still covers the reservoir vias so that no air is introduced into the vias.

90. Refresh the media reservoirs with fresh, equilibrated, EGM-2.
91. Perform a via wash, then return the trays of Pods to the Zoë and resume organ-chip culture.

### Ending organ-chip culture

**Timing: 10 min**

92. After 6 days in culture the chip experiment is ended.
93. Pause the Zoë and remove the trays and place in the BSC.
94. Inspect each chip for bubbles by eye.
95. Using a microscope, inspect cells in the chips to assess morphology and viability. If desired capture representative images at 10X or 20X magnification.
96. Remove the Pod lids and collect effluent medium from Pod outlet reservoirs for analysis, avoiding disturbing the Pod reservoir vias.
97. Gently aspirate any medium not collected for analysis, ensuring that a thin liquid film still covers the reservoir vias so that no air is introduced into the vias.
98. Choose which chips will be used for microscopy, which will be used for RNA isolation and which for macrophage flow cytometry.
99. Remove Chips from the pods and continue with the downstream processing.
100. There are several different analyses that can be performed on the chips, included in this paper are:
  a. Immunofluorescence imaging of whole chips.
  b. Flow cytometry of macrophages retrieved from collagen.
  c. qPCR on endothelial cells.
  d. qPCR on macrophages retrieved from collagen.
  e. Multiplex Secretome analysis of digested collagen.

### Chip fixation and staining for immunofluorescence

**Timing: 2.5 days. Chip fixation: 1 h 30 min. Chip staining: 2 days.**

This section describes the fixation of the cells in the chip and subsequent staining for imaging using fluorescence microscopy.

**CRITICAL:** Sufficient chip washing is vital for staining success. The wash steps should not be reduced. Longer washing is better than too short.

101. Ensure all chips are labeled and identify the different conditions clearly. Organize the chips in petri dishes for handling.

**CRITICAL:** Do not wash the chips before addition of formaldehyde.

102. Remove the PET disc from the inlet of the gel channel.
103. Loosely plug both top and bottom chip outlets, as well as the inlet of the gel channel with 200 μL non-filtered tips.
104. Using a 200 μL tip, take up 200 μL of 3.7% formaldehyde. Pipette slowly into the bottom channel, until about half of the formaldehyde has been pipetted in.
105. Leave the tip in the inlet. Let the remaining solution gravity flow into the top channel.
106. Add 200 μL of 3.7% formaldehyde slowly to the tip in the bottom channel inlet bringing the volume up to 400 μL.
107. Incubate for at least 1 h at room temperature (18°C–21°C) on a rocker.

***Note:*** Chips need to be on the rocker so that the fluid flows back and forth between the two ports and the two channels.

108. Whilst still on the rocker, remove the tips from all ports and replace the tips in the outlets of both channels, as well as the inlet of the gel filled channel with 200 μL tips.
109. Wash by adding 200 μL of PBS slowly to the bottom channel, leaving the tip in the inlet, letting the remaining solution gravity flow into the top channel.
110. Allow the chips to wash for 10 min before adding another 200 μL of PBS slowly to the bottom inlet tip, bringing up to 400 μL.
111. Allow the chips to wash for 10 min before adding another 200 μL of PBS slowly to the bottom inlet tips, bringing up to 600 μL.
112. Allow to wash for 10 min.

**Pause point:** Store at 4°C for up to 2 weeks. Do not allow chips to dry out as this can cause salt crystals from the PBS to block the channels.

113. Whilst still on the rocker, remove the tips from all ports and replace the tips in the outlets of both channels, as well as the inlet of the gel filled channel with 200 μL tips.
114. Permeabilize samples with 0.1% triton x-100 in PBS.
  a. Add 100 μL of this permeabilizing solution to the inlet of the endothelial channel.
  b. Leave pipette tips inserted in the ports.
115. Incubate chips for 30 min at room temperature (18°C–21°C) on the rocker.
116. After incubation remove all pipette tips and wash the channel with 200 μL PBS 3 times. As in steps 108–112.
117. Whilst still on the rocker, remove the tips from all ports and replace the tips in the outlets of both channels, as well as the inlet of the gel filled channel with 200 μL tips.
118. Block samples by incubating in 5% BSA in PBS.
  a. Add 100 μL of blocking buffer to the inlet of the endothelial channel.
  b. Leave pipette tips inserted in the ports.
119. Incubate for at least 1 h at room temperature (18°C–21°C).
120. After incubation remove all pipette tips and wash each channel with 200 μL PBS 3 times. As in steps 108–112.
121. Whilst still on the rocker, remove the tips from all ports and replace the tips in the outlets of both channels, as well as the inlet of the gel filled channel with 200 μL tips.
122. Prepare primary antibody solution(s) for each channel by diluting the desired primary antibodies in 1% BSA-PBS (Table 1).

**Table 1:** Antibody panel for staining chips

Target	Primary antibody	Dilution	Secondary antibody	Dilution
VE-Cadherin	Mouse monoclonal IgG_1_ anti-VE-Cadherin	1:100	Goat anti-Mouse IgG1 Alexa Fluor 647	1:200
CD64	CD64 Recombinant Rabbit Monoclonal Antibody (27)	1:100	Goat anti-Rabbit IgG (H + L) Alexa Fluor 488	1:200
CD68	CD68 antibody [KP1]( IgG1) or CD68 Monoclonal Antibody (514H12) (IgG2a)	1:100	Goat anti-Mouse IgG1 Alexa Fluor 647 or Goat anti-Mouse IgG2a Alexa Fluor 568	1:200
Nucleus	Hoechst	1:1000	-	-
123. After preparing the primary antibody solution(s), add 100 μL to the inlet of the endothelial channel, leaving pipette tips inserted in the ports.
124. Incubate chips for 4 h at room temperature (18°C–21°C) on the rocker.
125. After incubation remove all pipette tips and wash each channel with 200 μL PBS 3 times. As in steps 108–112.
126. Whilst still on the rocker, remove the tips from all ports and replace the tips in the outlets of both channels, as well as the inlet of the gel filled channel with 200 μL tips.
127. Prepare secondary antibody solution(s) for each channel by diluting the desired secondary antibodies in 1% BSA-PBS (Table 1).
128. Add 100 μL of the secondary antibody to the inlet of the endothelial channel, leaving pipette tips inserted in the ports.
129. Incubate chips overnight (minimum 12 h) at 4°C on the rocker, taking care to protect them from light.
130. After incubation, remove all pipette tips and wash each channel with 200 μL PBS 3 times. As in steps 108–112.
131. Whilst still on the rocker, remove the tips from all ports and replace the tips in the outlets of both channels, as well as the inlet of the gel filled channel with 200 μL tips.
132. Add 100 μL of 1:1000 Hoechst as a nuclear stain to the inlet of the endothelial channel, leaving pipette tips inserted in the ports. Incubate for 4 h at room temperature (18°C–21°C) in the dark.
133. After incubation, remove all pipette tips and wash each channel with 200 μL PBS 3 times. As in steps 108–112.
134. The chips are now ready to image. Store samples in the dark before imaging.

**Pause point:** Store samples in the dark at 4°C before imaging, do not allow chips to dry out. Image within 2 weeks.

### Immunofluorescence imaging

**Timing: 8 h**

***Note:*** In this paper the chips were imaged on the Leica Mica microscope system. Steps 135–141 are suggested settings.

135. Fluorescence Mode set to ‘Confocal’.
136. Fluorescence Setting split into sequential image acquisition for each fluorophore.
137. Intelligent Imaging.
  a. Field of View Frame Size set to Full Frame.
  b. Resolution set to Confocal Grade.
  c. Speed vs. Quality set to 2.4 s.
138. LIGHTNING on under Experiment Settings.
139. Capture images using the 10x objective to capture the full width of the channels.
140. To create high quality Z-stacks the ideal maximum thickness of each slice is 1 μm.
  a. The number of Z-slices can exceed 500.
141. To create the 3D rendered animations Imaris software was used (Videos S1 and S2).
  a. Surfaces were created based on size and z-position to separate the endothelial cells from the macrophages.
  b. Macrophages can be given a range of color dependent on the z-position.

***Alternatives:*** This protocol was optimized using the Leica Mica microscope; however, other imaging platforms and analysis software could be used following optimization.

Video S1. Representative 3D rendering of unstimulated whole chip related to step 141 Cyan: VE-cadherin (endothelium), Red: CD68 (pan-macrophage) Green: CD64 (pro-inflammatory macrophage) N = 3.

Video S2. Representative 3D rendering of whole chip with IL-1β treatment of the endothelium related to step 141 Cyan: VE-cadherin (endothelium), Red: CD68 (pan-macrophage) Green: CD64 (pro-inflammatory macrophage) N = 3.

### Vascular channel: Endothelial cell harvest for RNA isolation

**Timing: 15 min**

This step details the harvest of endothelial cells from the bottom channel. These cells can then be used to isolate RNA and to perform qPCR.

142. Rinse the bottom channel once by pipetting 200 μL of PBS through the channel inlet while aspirating the outflow.
143. Remove the PET disc from the inlet of the gel channel.
144. Plug the inlet and outlet of the channel opposite the channel of interest with empty 200 μL tips.

***Note:*** The channel of interest is the channel that contains the cells that are to be collected and lysed, in this case the bottom channel is of interest, so the top channel should be plugged.

145. Gently wash the channel of interest again with 200 μL PBS.
146. After washing, gently aspirate the PBS from the channel, leaving it dry.
147. Plug the outlet port of the channel of interest with an empty 200 μL tip.
  a. Ensure the tip is not pushed completely against the bottom of the channel to allow for smooth flow of accutase in and out of the pipette tip.
  b. At this stage there should be a total of three (3) tips inserted into ports of the chip.
148. Add 150 μL of accutase using a 200 μL tip into the channel of interest (through the inlet-port) and pipette up and down several times.
149. Plug the inlet port with the same 200 μL tip.
150. Allow cells to detach for 4–5 min in the BSC or 2–3 min in the incubator (37°C 5% CO_2_).
151. View the chip under the microscope to confirm cell detachment.
152. When cells are loose, reattach the pipette tip at the inlet-port of the channel of interest to the p200 pipette. Pipette up and down several times to thoroughly detach all cells.
153. Collect the cell suspension in an RNase-free Eppendorf tube.
154. Collect any remaining cells in the channel by washing with medium in the same manner as cells were detached.
  a. Pipette 150 μL of medium into the channel of interest.
  b. Pipette up and down several times.
  c. Collect in the same RNase-free Eppendorf tube.
155. Add 500 μL EGM-2 to the RNase-free Eppendorf tube to neutralize the accutase.
156. Spin the cells at 400g for 5 min at 21°C.
157. Aspirate EGM-2 using a p1000 pipette.

***Note:*** The pellet will be very small and difficult to see.

158. Add 300 μL of TRIzol and re-suspend the cell pellet. Alternatively, store the cell pellet at −70°C until use.
159. Follow your regular RNA isolation protocol from this point forward.

**Pause point:** The endothelial cell pellet or TRIzol lysed cells can be stored at −70°C for several months before RNA isolation.

### Collagen digestion and macrophage collection for RNA isolation or flow cytometry analysis

**Timing: 2–3 h**

In this step the collagen is digested to retrieve the cells in the plaque compartment. These cells can be lysed for RNA isolation or used fresh for phenotyping using flow cytometry.

***Note:*** The cells in the bottom channel should be removed prior to collagen digestion. This can be done as described in the section Endothelial cell harvest for RNA isolation.

***Note:*** Do not discard the digested collagen if you want to perform secretome analysis.

160. Warm up digestion solution (detailed recipe can be found in the materials and equipment section) in a 37°C water bath.
  a. 200 μL of digestion solution is needed for 1 chip.
161. Remove the PET disc from the inlet ports.
162. Plug the inlet and outlet of the top channel with pipette tips containing 50 μL digestion solution.
163. Plug the outlet of the bottom channel with an empty pipette tip. Plug the inlet of the bottom channel with a pipet tip containing 100 μL digestion solution.
164. Incubate the chip in the incubator (37°C 5% CO_2_) for 1 h.
165. Pipette the digestion solution up and down in both the top and bottom channels to loosen/break up the gel without removing the pipette tips.
166. Incubate in the incubator (37°C 5% CO_2_) for another 45 min.
167. Pipette the digestion solution up and down in both the top and bottom channels to loosen/break up the gel without removing the pipette tips.
168. Check for the presence of clumps, if necessary, incubate for an additional 30 min to break clumps.
169. If for RNA:
  a. Collect the collagen/cell mixture in an RNase free Eppendorf tube. Rinse the channels with PBS then spin the tube at 500g for 5 min at 21°C.
  b. Repeat for all chips.
  c. Aspirate PBS using a p1000 pipette. The pellet will be very small and difficult to see.
  d. Add 300 μL of TRIzol and re-suspend the cell pellet. Alternatively, store the cell pellet at −70°C until use.
  e. Follow your regular RNA isolation protocol from this point forward.

**Pause point:** The macrophage cell pellet or TRIzol lysed cells can be stored at −70°C for several months before RNA isolation.

170. If for flow cytometry:
  a. Collect volume from both channels in a well (use V- or U-well 96-well plate).
  b. Add 150 μL of cold FACS buffer and rinse both channels.
  c. Collect and add the volume to a second well.
  d. Check under microscope for removal of cells, repeat FACS buffer rinse if necessary.
  e. Repeat for all chips.
  f. Spin the plate at 400 g for 4 min at 4°C.
  g. Observe the wells under the light (focus on the walls of the wells) and aspirate the supernatant till the U of the well. Save digested collagen at this point.
  h. Wash well with 150 μL FACS buffer and centrifuge 400 g for 4 min at 4°C (pool wells if multiple wells per sample were used).
  i. Follow regular FACS staining protocol from this step.

### Flow cytometry panel and gating

**Timing: 1 h 30 min**

171. Measure samples on a suitable flow cytometer for the antibody panel detailed in Table 2.

**Table 2:** Antibody panel for flow cytometry staining macrophages from chips

Target	Fluorophore	Dilution
CD14	APC-H7	1:100
HLA-DR	PerCP-Cy5.5	1:200
CD11b	PE-Cy7	1:100
CD80	PE	1:100
CD86	FITC	1:100
CD64	APC	1:100
Live/Dead	DAPI	1:5000

**Table 3:** Setup of FACs Canto II

Laser wavelength	488nm	488nm	488nm	488nm	405nm	405nm	633nm	633nm
Filter	530/30	585/42	670LP	780/60	450/50	510/50	661/20	780/60

***Note:*** In this paper a FACS Canto II was used, the setup of the machine can be seen in Table 3.

**Figure 2:**
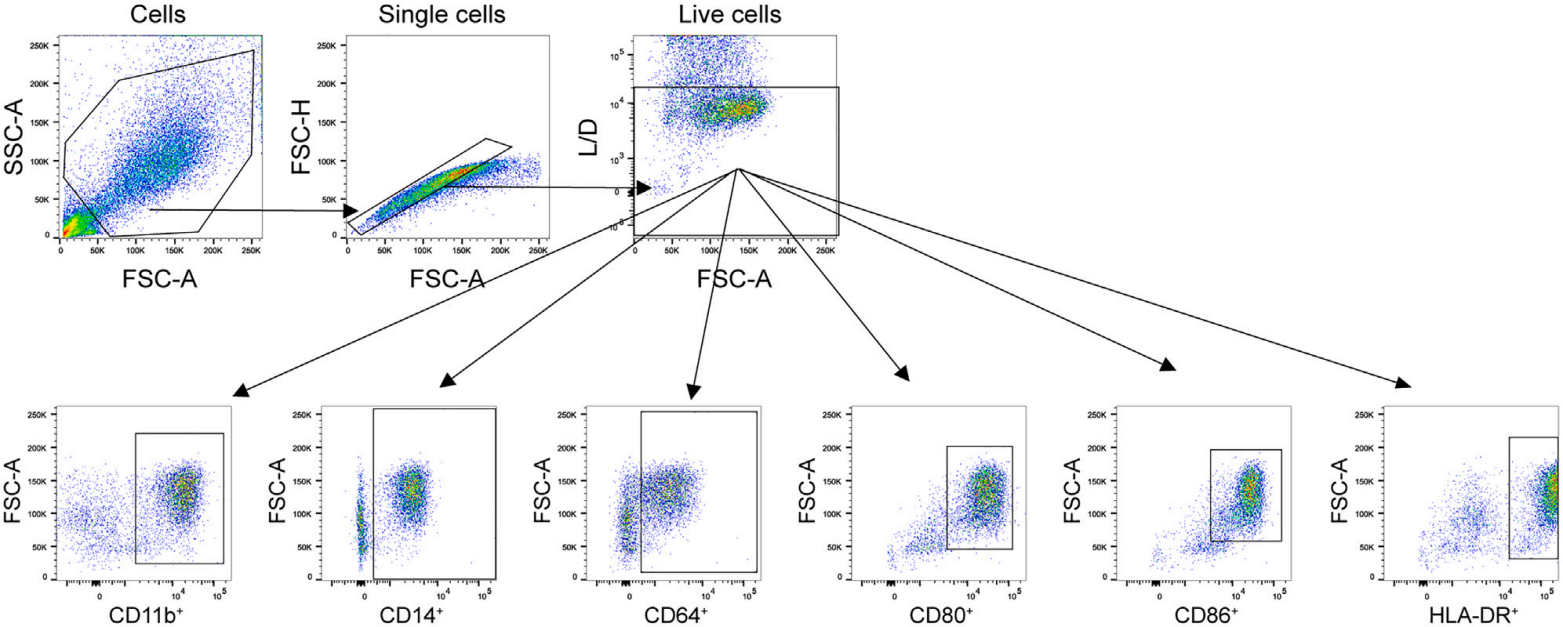
Flow cytometry gating strategy for macrophages retrieved from collagen Cells are gated on forward and side scatter area (FSC-A and SSC-A respectively), singlets are gated based on the FSC-A and forward scatter height (FSC-H). Then, live cells (DAPI^-^) are gated followed by gating of individual macrophage markers. *N* = 3.

172. Follow the gating strategy to analyze the expression of the various macrophage markers (Figure 2).

### qPCR

**Timing: 2 h 30 min**

qPCR of ligand-receptor pairs can be performed on the RNA obtained from the endothelial cells and macrophages. The list of primers used for endothelial cells can be found in Table 4. The primers used for macrophages can be found in Table 5.

173. Prepare the qPCR reaction master mix (Table 6).
174. Perform qPCR with the cycling conditions as in Table 7.

**Table 4:** Endothelial primers

Gene	Forward sequence	Reverse sequence
*36B4*	ACGGGTACAAACGAGTCCTG	GCCTTGACCTTTTCAGCAAG
*CCL2*	TGTCCCAAAGAAGCTGTGATC	ATTCTTGGGTTGTGGAGTGAG
*PLAUR*	CAGTGTAAGACCAACGGGGAT	CAGCTCTTCTCCTTCTTCCCAC
*MIF*	GCCCGGACAGGGTCTAC	CCTAGAACACAGCGTGCGG

**Table 5:** Macrophage primers

Gene	Forward sequence	Reverse sequence
*36B4*	ACGGGTACAAACGAGTCCTG	GCCTTGACCTTTTCAGCAAG
*CCR2*	CCA CAT CTC GTT CTC GGT TTA TC	CAG GGA GCA CCG TAA TCA TAA TC
*PLAU*	GCCACACACTGCTTCATTGAT	CTTCATCTCCCCTTGCGTGT
*CXCR2*	CAGGTGAAAAGCCCAGCGAC	GTAGAAAAGGGGGCAGGGTAG

**Table 6:** PCR reaction master mix

Reagent	Amount
cDNA template	3 μL (5 ng/μL)
Syber Green	5 μL
Primer (forward)	0.5 μL (10 μM)
Primer 2 (reverse)	0.5 μL (10 μM)
ddH_2_O	1 μL

**Table 7:** PCR cycling conditions

Steps	Temperature	Time	Cycles
Initial Denaturation	95°C	10 min	1
Denaturation	95°C	15 s	39 cycles
Annealing and Extension	60°C	30 s	
Melt curve	65°C–95°C in 0.5°C increments	5 s / 0.5°C	1
Hold	4°C	Forever	

### ELISA/multiplex array

**Timing: 1–2 days**

From the samples taken from the pod reservoirs ELISA or multiplex analysis can be performed. The digested collagen from the previous section can also be used to analyze the secretome of the macrophages in the collagen gel.

***Note:*** In this paper the Proteome Profiler Human XL Cytokine Array Kit was used to analyze the endothelial and macrophage secretome.

## Expected outcomes

### Flow cytometry

**Figure 3:**
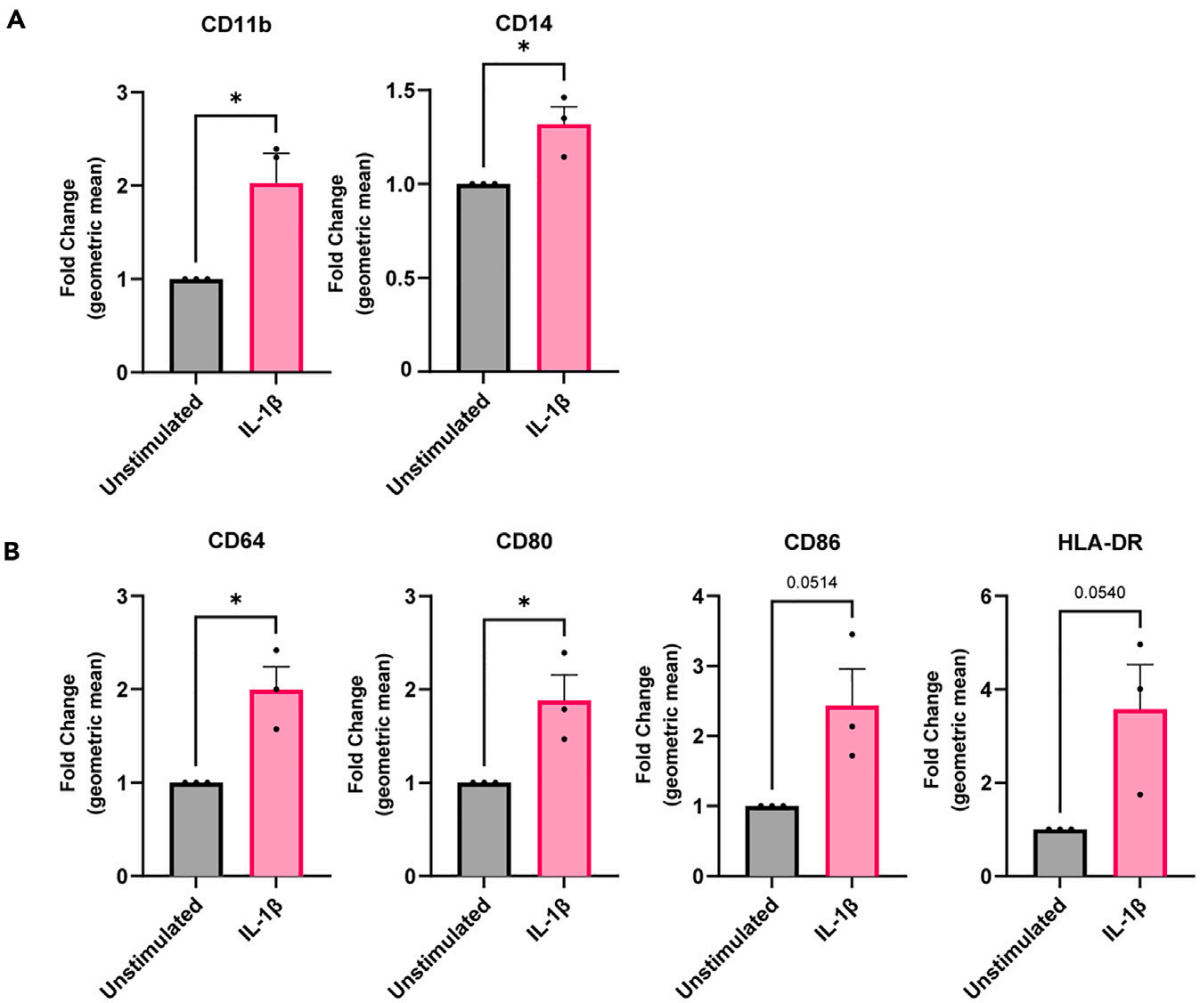
An activated endothelium results in inflammatory macrophages After treatment ± IL-1β of the endothelium (A) the pan-macrophage markers CD14 and CD11b and (B) the pro-inflammatory macrophage markers CD64 and CD80, CD86 and HLA-DR were measured using flow cytometry. *N* = 3, data are represented as fold change of the geometric mean compared to unstimulated. Error bars are SEM. ∗*p* < 0.05.

The average percentage of live cells collected by the flow cytometer is 69% (freq. of parent, see Figure 2 for the gating strategy). The expression of various (pro-inflammatory) macrophage markers with and without endothelial activation with IL-1β can be detected using flow cytometry (Figure 3). The pan-macrophage markers CD14 and CD11b are increased following endothelial activation (Figure 3A), as are the pro-inflammatory macrophages markers CD64, CD80, CD86 and MHC-II (HLA-DR) (Figure 3B).

### Immunofluorescence imaging

**Figure 4:**
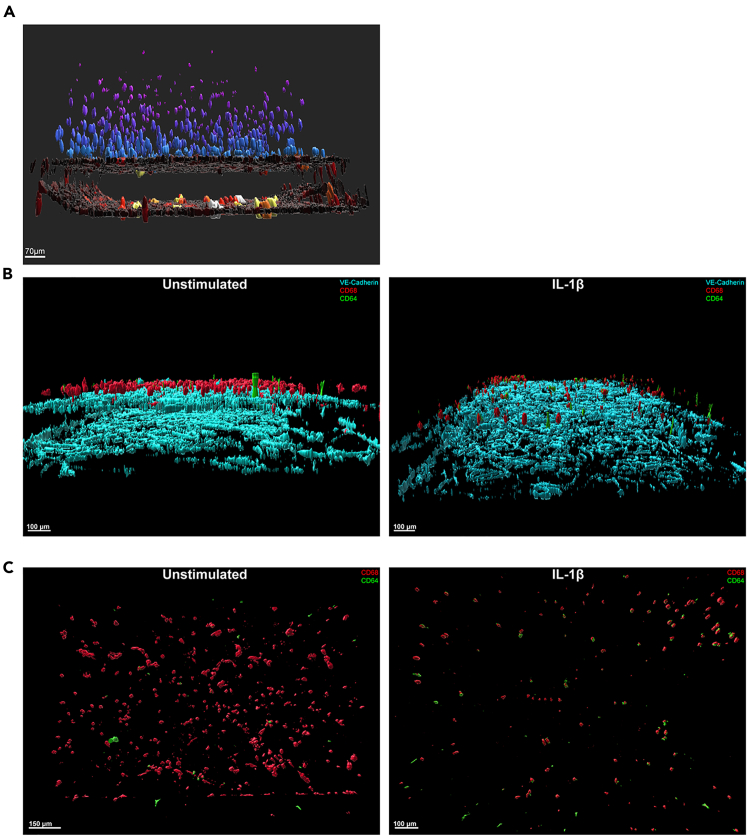
The pro-inflammatory marker CD64 is more highly expressed in chips with an activated endothelium Representative rendering in Imaris of a cross section of both chip channels. (A) Below in dark brown are the endothelial cells, above are macrophages. The color of the macrophages ranges from blue (close to the endothelium) to purple (further from the endothelium). Scale bar 70 μm. (B) Pro-inflammatory macrophage polarization in whole chips ± IL-1β treatment of the endothelium. Cyan: VE-cadherin (endothelium), Red: CD68 (pan-macrophage) Green: CD64 (pro-inflammatory macrophage). Scale bar 100 μm. (C) Representative rendering in Imaris of the top view of pro-inflammatory macrophage polarization in whole chips ± IL-1β treatment of the endothelium. Red: CD68 (pan-macrophage) Green: CD64 (pro-inflammatory macrophage). Scale bars 150 μm and 100 μm. *N* = 3.

To create the 3D rendered animations Imaris software was used (Videos S1 and S2). Additionally, surfaces were created and color intensities were assigned to macrophages based on their Z-position. Macrophages are found closer to the endothelium and become less dense higher up in the collagen (Figure 4A). There is an increased expression of the pro-inflammatory macrophage marker CD64 (green) when the endothelium has been activated with IL-1β (Figure 4B).

### Secretome analysis

The secretome of the cells in the chips was measured using the Proteome Profiler Human XL Cytokine Array Kit on digested collagen. The digested collagen was diluted 5x in Array Buffer 6. Upon imaging on a Bio-Rad ChemiDoc system, the exposure was initially set to take one image each minute between 1 and 10 min. Following this, the exposure time was set to 20 min, and the image created was used for further analysis.

**Figure 5:**
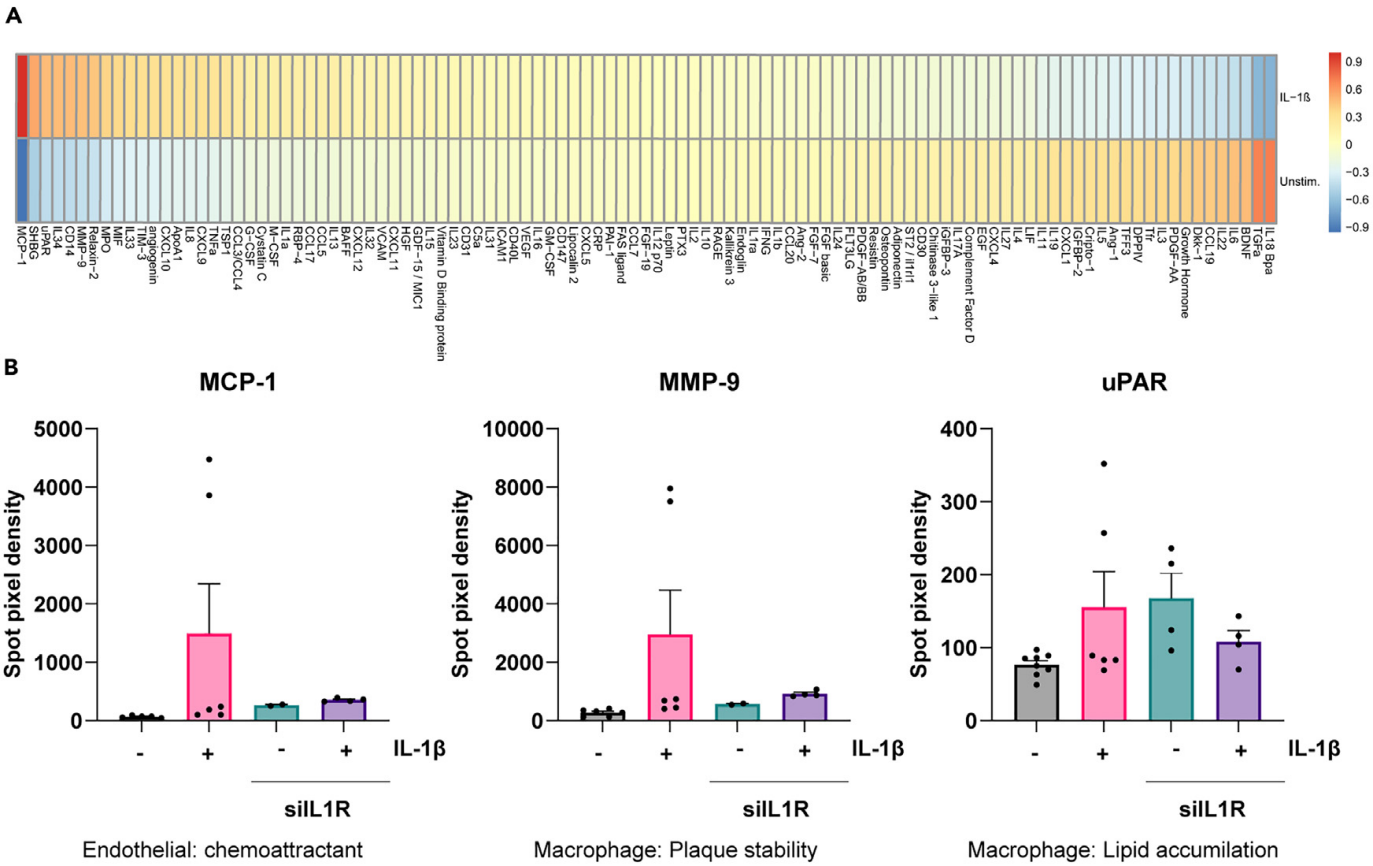
Activated endothelial cells result in an increased pro-inflammatory plaque microenvironment (A) Example heat-map of cytokines in digested collagen from chips with unstimulated or IL-1β stimulated endothelium. Cytokine intensities between unstimulated and IL-1β stimulated endothelium are displayed as a proportion and as colors ranging from red to blue as shown in the key. (B) Examples of differentially secreted cytokines and their role in atherogenesis. *N* = 3. Error bars are SEM.

The XL cytokine array can measure 105 cytokines and could potentially be used as a high throughput, unbiased screen before focusing on specific targets. For example, using a proportion heat-map (Figure 5A), our data show that the secretion of cytokines accumulating in the collagen varies between chips containing an unstimulated or activated endothelium. The spot pixel density of a selection of cytokines is also included (Figure 5B).

### qPCR

As only 200,000 HAECs and 350,000 monocytes are seeded in one chip this inherently low cell number also results in a low RNA yield. Therefore, it is advisable to pool 2 chips. When pooling 2 chips you can expect an RNA yield of approximately 35 ng/μL or 700 ng total RNA for the endothelial channel and 40 ng/μL or 800 ng total RNA for the macrophage channel.

**Figure 6:**
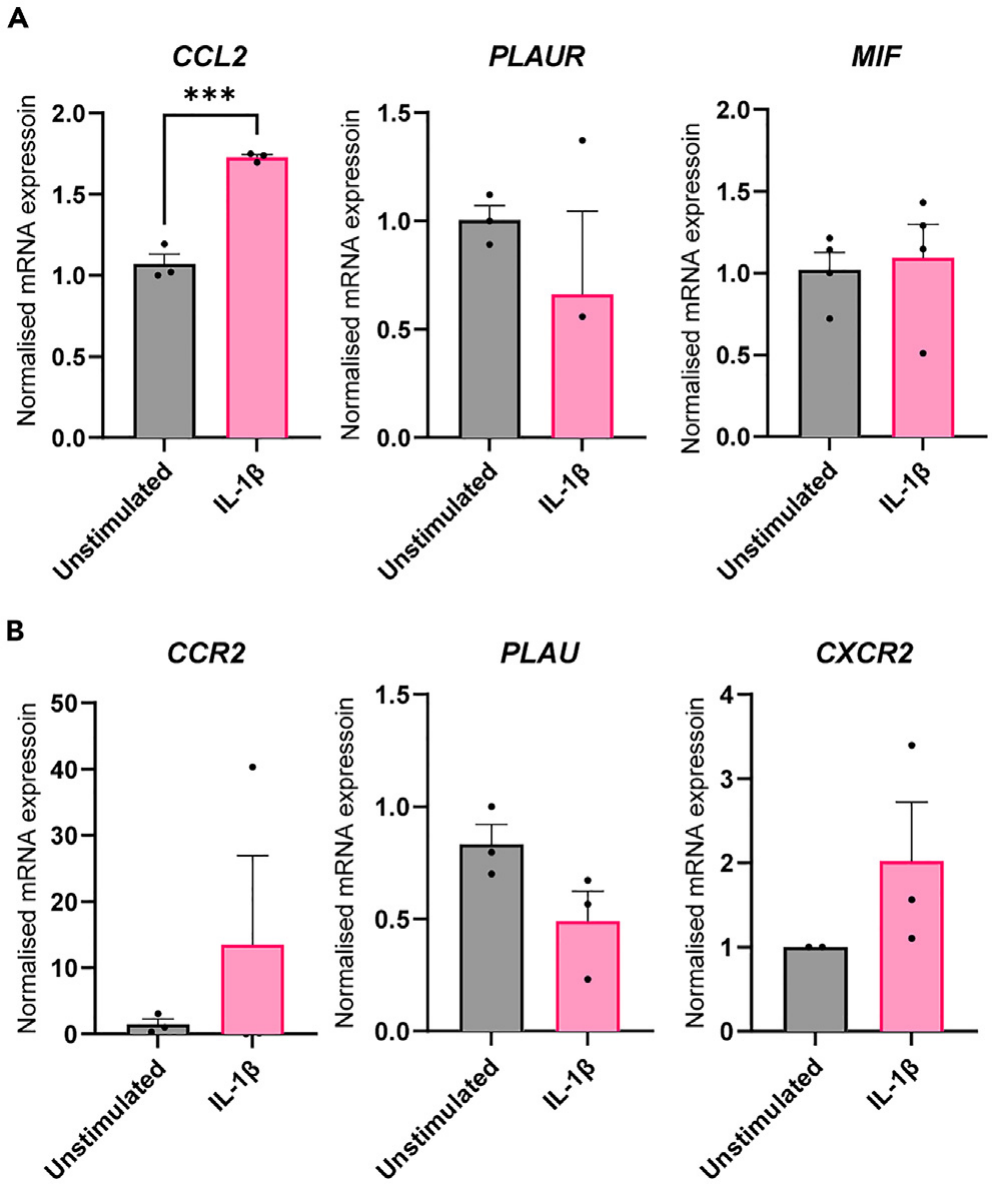
Ligand-receptor pairs can be measured using qPCR The expression of 3 L-R pairs was measured using qPCR. (A) Endothelial expressed *CCL2, PLAUR* and *MIF*. (B) Macrophage expressed *CCR2, PLAU* and *CXCR2*. *N* = 3. Error bars are SEM. ∗∗∗*p* < 0.001

Based on the secretome analysis, targeted ligand-receptor (L-R) pairs can be measured on qPCR (Figure 6). Alternatively, L-R interactions could be analyzed using scRNA-seq. This has not been done in this protocol.

The endothelial *CCL2*, *PLAUR* and *MIF* (Figure 6A) and their macrophage counterparts *CCR2*, *PLAU*, and *CXCR2* (Figure 6B) were measured in this targeted qPCr analysis. The mRNA expression patterns can be well replicated in both endothelial cells and macrophages.

### IL1R knockdown in HAECs

In order to confirm that it is the endothelial crosstalk inducing the pro-inflammatory polarization of the macrophages in the chip and not simply the IL-1β added to the system, an IL1R knockdown in the endothelial cells was performed.

**Figure 7:**
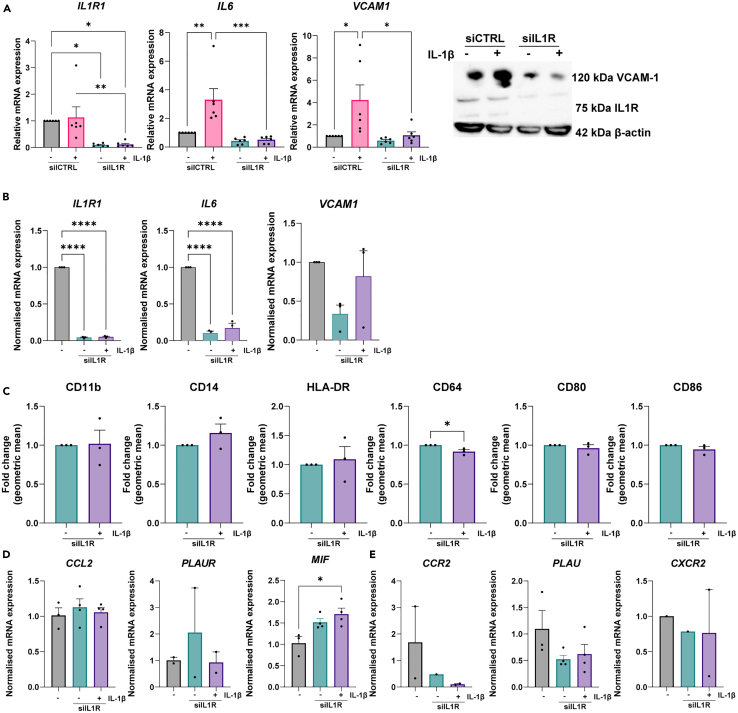
IL1R knockdown is successful and macrophages do not become pro-inflammatory if endothelial cells have IL1R knockdown (A) mRNA and protein expression of IL1R and downstream inflammatory markers IL-6 and VCAM-1 following siRNA treatment, *N* = 6. (B) IL1R, IL-6 and VCAM-1 mRNA expression at time of IL-1β stimulation in chip *N* = 3. (C) Expression of pan-macrophage markers CD14 and CD11b and pro-inflammatory macrophage markers CD64 and CD80, CD86 and HLA-DR after treatment ± IL-1β of the endothelium ± IL1R knockdown, measured by flow cytometry. Data are represented as fold change of the geometric mean compared to unstimulated. The expression of 3 L-R pairs was measured using qPCR. (D) Endothelial expressed *CCL2, PLAUR* and *MIF*. (E) Macrophage expressed *CCR2, PLAU* and *CXCR2*. *N* = 3. ∗*p* < 0.05, ∗∗*p* < 0.01, ∗∗∗*p* < 0.001. Error bars are SEM.

First, the IL1R knockdown was validated in 2D cell culture to confirm its efficacy before application in 3D chip culture. Validation in 2D cell culture demonstrates that the IL1R knockdown is successful in HAECs (Figure 7A) and endothelial cells do not become inflammatory following IL-1β stimulation (Figure 7A).

Next, chips were seeded with siIL1R endothelial cells. Additionally, ECs containing the IL1R knockdown were also plated in 2D culture to serve as a control. The 2D cultured control cells confirm that at the time of stimulation with IL-1β in the chip the IL1R knockdown was still active and the endothelial cells did not become pro-inflammatory following IL-1β stimulation (Figure 7B). After one week of chip culture, the macrophages retrieved from the collagen do not differ in pan-macrophage markers CD14 and CD11b expression when the endothelial cells had been stimulated with IL-1β or remained unstimulated (Figure 7C). Additionally, the macrophages do not become polarized toward a pro-inflammatory state as the expression of CD64, CD86 and CD80 are not different in the stimulated condition compared to the unstimulated condition (Figure 7C). The secretome of both the unstimulated and stimulated knockdown endothelium is similar to that of the unstimulated endothelium without knockdown (Figure 5B). Additionally the expression of various L-R pairs from the silenced endothelium have expression patterns similar to that of the unstimulated non-silenced L-R pairs (Figures 7D and 7E).From these results we can conclude that endothelial crosstalk is responsible for inducing the pro-inflammatory polarization of the macrophages in the chip. Therefore, this protocol is suitable for studying endothelial interventions on macrophage polarization.

## Quantification and statistical analysis

To quantify the mean spot pixel density of the Human XL Cytokine Array the DotBlot_Analysis.ijm^12^ macro was used in Image J. Then to create the heat map the proportion of the mean spot pixel density for the unstimulated and stimulated condition were plotted against each other, i.e., $\frac{\text{Unstimulated}}{\text{Unstimulated}+\text{IL}-1\beta }+\frac{\text{IL}-1\beta }{\text{IL}-1\beta +\text{unstimulated}}=1$. The absolute spot pixel densities were also plotted in bar graphs. Statistical analysis was performed in GraphPad Prism software. Comparisons between two groups were tested for statistical significance using an unpaired two-tailed *t*-test. Comparisons between 3 or more groups used a one-way ANOVA with multiple comparisons.

## Limitations

### Cell type

In this specific setting the chip protocol has been optimized and validated for using HAECs and fresh CD14^+^ bead isolated monocytes from human PBMCs. If the user wishes to use other endothelial cell types or immune cells, they will have to validate and optimize this. In particular the cell number and number of days required for a full confluent vessel channel to form can vary per cell type.

### Simplistic design

We are aware that this model only demonstrates a simplified plaque microenvironment, as an atherosclerotic plaque is comprised of more cell types than only macrophages. However, in the current context this allows for studying the direct interaction between the endothelium and macrophages. The model could be expanded to include additional cell types in the plaque compartment.

### Limited material

The number of cells that are seeded in a chip is low, namely 200,000 endothelial cells and 350,000 monocytes. The number of monocytes seeded within the collagen (as well as the collagen concentration) has been extensively optimized to retain the collagen integrity, whist maintaining imaging quality for immunofluorescence microscopy and cell retrieval for flow cytometry. This means that for certain assays, for example RNA isolation, the yield for the endothelial cells and macrophages is also relatively low. Currently we have been able to successfully perform qPCR analysis with satisfactory melt curves. If the yield is too low for a certain assay, multiple chips can be pooled together. This will mean more chips are used per experiment.

## Troubleshooting

For general troubleshooting tips regarding chip culture see Emulate’s troubleshooting guide (https://emulatebio.com/).

### Problem 1

Bubble formation causes organ-chip culture to fail.

There are several critical steps where bubble formation can affect organ-chip culture.

• During chip activation bubbles will prevent activation at that spot, meaning cells will not survive there.
• During chip coating bubble will prevent coating at that spot, meaning cells will preferentially not attach there.
• During seeding bubbles will prevent media flow and can impact cell attachment and survival.
• During chip culture bubble prevent media flow and can impact cell survival.

The following three steps not only affect cell survival, but can also damage the Zoë culture module.

• Incorrect gas equilibration of media will cause bubbles.
• Incorrect priming of pods can introduce bubbles.
• Removing all media when replenishing media in the pod reservoirs can introduce bubbles.

### Potential solution

At any stage if bubbles are present in the chip channels or ports wash the channel with the appropriate solution (activation solution, coating solution, media). If bubbles persist aspirate the channel until dry and then slowly reintroduce the solution.

If the medium takes longer than 10 s to pass through the Steriflip unit, gas equilibration is not sufficient. The vacuum pressure must be at least −70 kPA.

Check the underside of the pods after priming. If there are no media droplets then priming has failed. Run the prime protocol again.

### Problem 2

When preparing the collagen gel and monocyte mixture or when pipetting the collagen mix into the top channel the collagen already starts to polymerize.

### Potential solution

Work on ice, keep all reagents and cell mixtures on ice. Use frozen tips and Eppendorf tubes as well as any other material you use.

### Problem 3

The collagen gel lets loose from the top channel.

### Potential solution

We have performed extensive optimization to find the optimal collagen concentration in order for the gel to remain stable, but also not be too dense for imaging studies. The current concentration allows for chip culture for 7 days only. If users wish to culture for longer than 7 days they will have to optimize this. For example additional coating of the chip channel, for example with fibronectin, could allow the gel to adhere better to the chip channel. Alternatively, if no imaging studies are required the concentration of collagen could be increased.

### Problem 4

The background when imaging the chips can be quite high. This problem is not completely unavoidable, however by following the potential solutions the background can be minimized.

### Potential solution

Ensure the chips are washed for a long enough period of time after primary and secondary antibody incubations. In addition, the use of a rocker is essential to allow the wash to permeate through the gel. Do not vortex fluorophore conjugated secondary antibodies before use and pipette from the top of the antibody vial. This ensures any fluorophore sediment remains in the antibody vial. If possible use lightning deconvolution software to reduce background and enhance the signal from the cells.

### Problem 5

The target signal when imaging chips can be low.

### Potential solution

Validate antibodies on 2D cells first. Usually, the antibody concentrations needed in a chip are double that used on 2D samples. If possible use lightning deconvolution software to reduce background and enhance the signal from the cells.

### Problem 6

The number of cells that are seeded in a chip is low (200,000 HAECs and 350,000 monocytes). This means the RNA yield for the endothelial cells and macrophages is also relatively low.

### Potential solution

If the RNA yield is too low 2 chips can be pooled.

## Resource availability

### Lead contact

Further information and requests for resources and reagents should be directed to and will be fulfilled by the lead contact, Jeffrey Kroon, j.kroon@amsterdamumc.nl.

### Technical contact

Technical questions on executing this protocol should be directed to and will be answered by the technical contact, Jeffrey Kroon, j.kroon@amsterdamumc.nl.

### Materials availability

This study did not generate new unique reagents, all reagents are commercially available.

### Data and code availability

No datasets or codes were generated in this study.

## Acknowledgments

J.K. was supported by the Dutch Heart Foundation (Senior Scientist Dekker grant [03-004-2021-T045]) and funded by the European Union (ERC, ENDOMET-STEER, 101076407). Views and opinions expressed are however those of the author(s) only and do not necessarily reflect those of the European Union or the European Research Council Executive Agency. Neither the European Union nor the granting authority can be held responsible for them. Graphical abstract was created using Biorender.com. We are grateful for the excellent technical expertise from Emulate (Emulate, Boston, MA, USA). We would like to thank the Cellular Imaging and Flow Cytometry Core Facility at the Amsterdam UMC, location AMC, for their excellent technical support in this study. We would like to thank Dr. Bosmans, X. Zhang, S. van Kesteren, M.C. Peletier, and T.J. van Driel for critically reading the first draft of the manuscript. Finally, we would like to thank the volunteers who donated blood for this study.

## Author contributions

K.M.L.H. and J.K. conceptualized and developed the described protocol. K.M.L.H., K.E.D., and M.V. carried out the experiments. K.M.L.H. and J.P. analyzed the data. J.K. provided input through scientific discussion of the experimental data. K.M.L.H. wrote the first draft of the manuscript. All authors provided constructive and critical feedback on the manuscript draft. Funding was provided by J.K.

## Declaration of interests

The authors declare no competing interests.
